# Identifying influential regions in extremely rare variants using a fixed-bin approach

**DOI:** 10.1186/1753-6561-5-S9-S3

**Published:** 2011-11-29

**Authors:** Michael Agne, Chien-Hsun Huang, Inchi Hu, Haitian Wang, Tian Zheng, Shaw-Hwa Lo

**Affiliations:** 1 Department of Statistics, Columbia University, 1255 Amsterdam Avenue, Room 1005, MC 4690, New York, NY 10027, USA; 2 Department of Information Systems, Business Statistics and Operations Management, Hong Kong University of Science and Technology Business School, Clear Water Bay, Kowloon, Hong Kong

## Abstract

In this study, we analyze the Genetic Analysis Workshop 17 data to identify regions of single-nucleotide polymorphisms (SNPs) that exhibit a significant influence on response rate (proportion of subjects with an affirmative affected status), called the affected ratio, among rare variants. Under the null hypothesis, the distribution of rare variants is assumed to be uniform over case (affected) and control (unaffected) subjects. We attempt to pinpoint regions where the composition is significantly different between case and control events, specifically where there are unusually high numbers of rare variants among affected subjects. We focus on private variants, which require a degree of “collapsing” to combine information over several SNPs, to obtain meaningful results. Instead of implementing a gene-based approach, where regions would vary in size and sometimes be too small to achieve a strong enough signal, we implement a fixed-bin approach, with a preset number of SNPs per region, relying on the assumption that proximity and similarity go hand in hand. Through application of 100-SNP and 30-SNP fixed bins, we identify several most influential regions, which later are seen to contain some of the causal SNPs. The 100- and 30-SNP approaches detected seven and three causal SNPs among the most significant regions, respectively, with two overlapping SNPs located in the *ELAVL4* gene, reported by both procedures.

## Background

The completion of the Human Genome Project in 2003 and the International HapMap Project in 2005 gave researchers access to exciting new technological tools in understanding human genetics, including large available databases. This new level of information has led to efforts to better understand the relationship between genetics and disease. At the forefront of these efforts are the genome-wide association studies, methods that rely on large-scale scanning of the association between genetic variants and particular diseases [1]. Since 2005, these studies have led to a better understanding of genetic susceptibility to more than 40 common diseases, including type II diabetes, Parkinson’s disease, and prostate cancer [2].

Even before 2005, many methods had been developed to reconcile genetic variants and vulnerability to disease. The common disease/common variant (CDCV) hypothesis, which suggests that “common variants may hold the secrets to many disease susceptibilities” [2], has been a long-held principle. Under this theory, a list of common SNPs and association mapping can be used to better understand which mutations are related to disease susceptibility. In essence, the genetic factors underlying common diseases will be alleles that are themselves quite common in the population at large [3].

According to the *American Journal of Psychiatry* in 2009, common SNP variants are by convention defined to vary “on at least 5% of chromosomes in the population” [1]. The CDCV strategy suggests that many different common SNPs have small effects on each disease and that some could be found by testing enough SNPs and subjects [2].

Recently, more attention has been given to the possible influence of rare variants on this same disease susceptibility [4]. A rare variant is defined by a frequency of less than 1% [5]. Although the methods used in genome-wide association studies (GWAS) currently struggle to powerfully address these potential rare SNP associations, efforts such as the 1000 Genomes Project may extend systematic GWAS methods to the 1–5% frequency range [2]. Blangero et al. [6] recently suggested that “uncommon or rare genetic variants can easily create synthetic associations that are credited to common variants” and called for follow-up in future GWAS studies. This field of rare variants will be the focus of this project.

It should be noted that the methods proposed were developed and implemented without prior knowledge of the true causal SNPs or of the model generation process. However, after implementation, we use this information to evaluate the success of our procedure.

## Methods

### Data set

Genetic Analysis Workshop 17 (GAW17) contains a simulated data set of 3,205 autosomal genes with 24,487 single-nucleotide polymorphisms (SNPs) genotyped on 697 subjects. The 1000 Genomes Project provided SNP genotypes for their pilot3 study. SNPs with missing data are imputed by fastPhase. Most of the allele frequencies for these markers are rare with a minor allele frequency (MAF) less than 1% (i.e., about 74%). Three quantitative trait values (Q1, Q2, and Q4) and affected status are generated in each simulated unrelated-individual data set. Affected status and genotype information are the focus of our case-control study. Two hundred simulations are conducted, each containing 209 affected cases and 488 unaffected control subjects. The genotypes are fixed for the 200 simulation replicates; however, the model-dependent phenotypes are different among simulations [7].

### Prominence of private SNPs in the data

A simple exploratory analysis of the SNPs at hand shows that the number of mutant alleles is heavily skewed toward low numbers. In fact, there are more private variants (9,433) than there are common variants with MAF > 1% (6,356). This motivates the exploration into these extremely rare variants, because they make up such a large proportion of the data.

### Collapsing of SNP information in the data

Because any one given private SNP can have only a limited influence on the data, we must use some sort of collapsing method (a way to combine the information of several SNPs) to attain any sort of power for association testing. Several methods have been proposed to achieve this end. SNPs from the same genes could be combined; the same could be done for SNPs from the same pathway of genes, or even an arbitrary chromosomal region defined by base-pair positions on a chromosome [7]. However, both of these methods have procedural limits. The genes vary in number of SNPs contained, and 38% of genes in the data contain only one SNP, meaning no information would be merged within those regions. Moreover, 68% of genes contain fewer than five SNPs, meaning the enhancement of signal would be extremely limited. Further, use of pathways would alleviate some of these problems but would also require extra outside information, which would further complicate our procedure.

### Collapsing method: fixed bins

We implement a straightforward approach to collapse several SNPs in the data. We order the SNPs and divide them into a chosen number of fixed bins. We rely on the assumption that SNPs in close proximity to each other within a chromosome will exhibit some level of association. This allows for many of the SNPs within the same genes to be grouped together and permits a combination of nearby, smaller genes to merge their respective information, which would otherwise be difficult to extract. This also adds the bonus that each fixed bin is of equal size.

We have both biological and genetic reasons for binning fixed numbers of SNPs. Our purpose is not to identify individual SNPs but to specify localized regions that contain candidate SNPs. We first attempted the method with a gene-based approach but found that the results were quite weak, mainly because many of the genes were relatively short and there were few private SNPs within each gene. With larger regions, we have more private SNPs in general within each gene, allowing us to detect a significant result. Within a chromosome, nearby SNPs may have some association through linkage disequilibrium. Merging the information of nearby genes will allow us to accentuate a signal by incorporating nearby associated SNPs, even if they are not biologically causal themselves. Our fixed-bin method also ensures that regions are nonoverlapping. Although this is not the case in our simulated data, it is possible that some SNPs may overlap more than one gene (or chosen genetic region), inflating type I error, or that some may fail to belong in any region (guaranteeing that they will not be identified). The fixed-bin approach allows a simple, computationally efficient method for enhancing signal.

In this study, we execute a fixed-bin approach with bin sizes of 100 and 30 SNPs, respectively. We have determined that our procedure is quite robust to any bin size between these two numbers.

### Difference in proportions between case and control groups: extreme rare variants

We first divide all the SNPs into regions. Although we rely on proximity within a chromosome to merge data, we prohibit regions from containing SNPs from different chromosomes. To this end, we cut off regions at the end of each chromosome. For the 100-SNP window approach, we have 253 regions. Those regions at the end of the chromosome are either left smaller than 100 SNPs or, if they are below a certain threshold of length (in this case we set the threshold at 25 SNPs), they are merged with the previous region to create a slightly larger one. An analogous approach is used with a fixed-bin size of 30 SNPs with 816 regions. Within each region, we are interested in the ratio of extreme rare variants (i.e., only one allele difference in the SNP) and in the difference of this value from those that fall into the case and control categories. We focus only on affected status; thus we define the *case* group as those with affected status = 1 and the *control* group as those with affected status = 0. Within each region we define the following value:(1)

where *i* = 1, …, number of fixed bins.

### Significance calculated by permutation test

Our method can be easily extended to analyze rare SNPs that are rare but not private (i.e., a few minor alleles). In our analysis, we focus on private variants. We use a standard nonparametric permutation test to determine whether the proportion of extreme variants in case subjects is significant. To do so, we take the SNPs from each interval and randomly permute the affected status. We then define the permutated *p*-value for fixed bin *i* as the number of {observed *p_i_* < permuted *p_i_*}/1,000. That is, those regions where the observed *p_i_* is significantly higher than usual will have a low *p*-value, thus demonstrating significance. If only one simulation were available, we would set a cutoff for the *p*-value (5%) and report those regions with *p-*value less than 5% to be potentially significant regions. However, if we use more than one simulation, we must find a way to combine these simulation-by-simulation reports of significant regions.

### Aggregation of significance through all 200 simulations

We propose a method to order intervals by significance. In this case, we wish to report which regions are relatively *most significant*. We empirically report the 10 most significant regions, with the assumption that this might narrow down future analysis by the scientist. To gauge significance, we define the term *return frequency*, which is simply a count of the number of times a region is significant over the 200 simulations.

Although the *p-*value threshold for each of the simulations is 0.05, in order for a region to be returned as one of the most significant regions in our results, it must pass this threshold many times. In fact, all of our top 10 regions exceed this threshold by more than 50 times. A Poisson estimate of this probability is: 10^50^*e*^–10^ / 50! ≈ 10^–19^.

## Results

**Figure 1:**
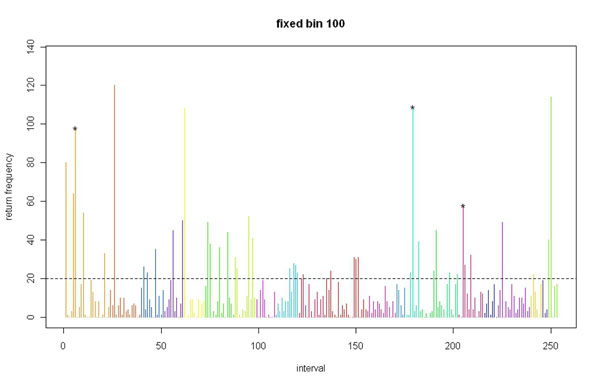
Significance return frequency by region using the 100-SNP fixed-bin approach.

**Figure 2:**
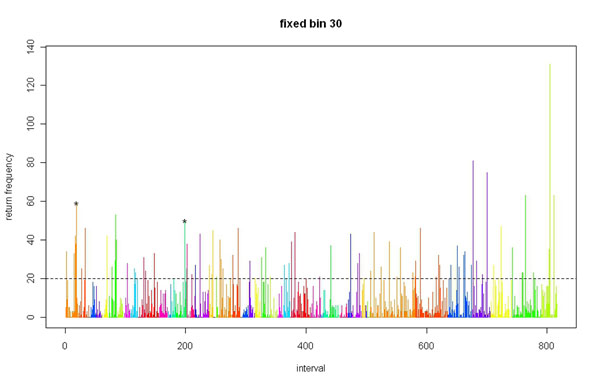
Significance return frequency by region using the 30-SNP fixed-bin approach.

Figures 1 and 2 summarizes the return frequency for all regions, significant or not, for both the 100- and 30-SNP approaches.

Based on the *p-*value threshold of 0.05, the expected return frequency for a certain region in the 200 simulations is about 10 (i.e., 200 × 0.05). As approximated by the Poisson distribution, a return frequency greater than 20 is almost three standard deviations away, which is an extreme situation. The top 10 return regions listed for both the 30-SNP and 100-SNP fixed bins show return frequencies greater than 50.

### 100-SNP fixed-bin approach with return frequency

The results for the return frequency are shown in Table 1. We order the top 10 most significant regions and determine that three of them contain causal SNPs. Region 179 contains the gene *SOS2* (causal SNP C14S1381), and region 6 contains three causal SNPs (C1S3181, C1S3182, C1S2919). Also in the top 10 was the region containing the gene *SREBF1* (10 causal SNPs, 3 of which are private [C17S1009, C17S1030, C17S1056]).

**Table 1:** Return of the top 10 regions (fixed bin = 100)

Region	Return frequency	True SNP
26	120	
250	114	
179	109	C14S1381
62	108	
6	98	C1S3181, C1S3182, C1S2919
1	80	
5	64	
205	58	C17S1009, C17S1030, C17S1056
10	54	
95	52	
805	131	
677	81	
701	75	
764	63	
811	63	
19	59	C1S3181, C1S3182
84	53	
199	50	C4S4935
724	47	
33	46	

### 30-SNP fixed-bin approach with return frequency

In Table 1, we list the top 10 most significant regions, and two of them contain causal SNPs. One of these regions contains two causal SNPs (C1S3181, C1S3182) that were also identified by the 100-SNP fixed-bin approach. Another region contains the gene *VEGFC*, which contains one causal SNP (C4S4935). This is particularly promising because this gene contains only that one SNP.

## Discussion

Although many methods can pinpoint influential common variants, there has been a widespread struggle to pinpoint influential private variants. The promise of our results lies in the fact that we are able to identify regions that do indeed contain several causal private variants. Although seven and three causal SNPs are not huge numbers, by a conservative estimate in which we assume that all causal private SNPs are uniformly distributed throughout regions, we would expect our 10 most influential regions out of the 253 and 816 regions in the 100-SNP and 30-SNP fixed-bin approaches, respectively, to show 90(10/253) ≈ 3.56 and 90(10/816) ≈ 1.10 out of the 90 true causal private SNPs. Moreover, we can almost be sure that a gene-based approach would struggle to pick up the causal SNP C4S4935 in *VEGFC*, which is composed of only one SNP.

Of course, there are several drawbacks to these results. First, even without knowledge of the causal SNPs, we know that our identification of influential regions takes us only as far as the region at hand. It does not allow us to pinpoint exactly which SNPs within this region are influential. However, as noted before, the narrowing down of potential regions can save countless time and money for the scientist. A region here contains 100 or 30 SNPs, as opposed to the approximately 25,000 in the complete data set.

Second, our motivation is that we are anticipating several causal rare SNPs in the same gene and in several patients. Suppose that variants in different genes are responsible. Then methods that incorporate interaction between genes are needed to detect it. This will boost power significantly. However, we see numerous false positives in our results. In fact, the majority of our results show up as false positives. To a certain extent, this can be expected, because the influence of any one private variant is small and it is quite difficult to determine causality when private variants are so sparse. Expectations have to be tempered when dealing with such private variants. Furthermore, the specific data set at hand may be specifically troubling for this procedure, although this same struggle may not be imitated in real data. For all 200 simulations, the same genotype is used. Thus it may be possible that any association by chance may be propagated through all 200 simulations. In essence, this “false positive” may indeed be a real signal from the data, just not an intended one. In fact, an exploratory analysis shows that many false-positive SNPs highly correlate with causal SNPs, so it is no surprise that they show up in the results.

In our approach, we report the results of fixed bin sizes of 30 and 100, although our procedure can be generalized to any size bin. Choosing a bin size much lower than 30 will aggregate too little information because too few private SNPs will be contained within each region to begin with. Choosing a bin size much larger than 100 will sacrifice specificity for the scientist when a much larger region of influence is identified with many more candidate causal SNPs; it will also lead to the possibility of variation contaminating the signal. We have implemented our method for bin sizes between the two extremes (e.g., with a bin size of 50) and have seen similar results, suggesting that our procedure is robust to any choice of size within this range.

Although the simplicity of our method means that we do not directly address such issues as density of markers throughout the chromosome, we can recommend that the choice of bin size be varied within this acceptable range based on such knowledge.

Although our analysis is nonparametric in the sense that it is based on “relative” significance of regions, we could attempt to implement an absolute cutoff for significance. This would help answer the question of whether there any significant regions, as opposed to the question we answer: Which regions have the greatest potential to be significant?

## Conclusions

The scientific community has had difficulty pinpointing influential private variants, but our procedure attempts to solve this challenging problem, with some degree of success. We observe that using a fixed-bin approach is sometimes more effective than groupings based on genes, some of which contain only one SNP, at least in this particular data set. At the same time, the method relies less on outside information than analyses based on gene pathways do.

In the end, our procedure reaches the roadblock of numerous false positives, although some may be inherent risks of the method of data generation in play. Nevertheless, our procedure retains its generality to many types of data sets, not just the one at hand. Our results with respect to these data are not mind-blowing, but this performance is specific to this data set. Knowing the linear regression nature of the data generation, one could implement simpler and most likely more effective procedures. However, in real life this knowledge is not available, and we attempt to design a procedure that can perform on any type of data set, not one that simply performs the best on this simulated one.

Ultimately, the fixed-bin approach we describe here offers a method whose value is its simplicity and ease of implementation in the face of a challenging problem.

## Competing interests

The authors declare that they have no competing interests.

## Authors’ contributions

SHL, MA, and CHH designed the study. MA, CHH, SHL, and TZ performed the study. MA, CHH, IH, HW contributed to analysis of the data. MA, CHH, and SHL drafted the manuscript. All authors read and approved the final manuscript.
